# Case report: paravalvular leak as a complication of percutaneous catheter ablation for atrial fibrillation

**DOI:** 10.1186/s13019-014-0187-4

**Published:** 2014-12-17

**Authors:** Orcun Gurbuz, Abdulkadir Ercan, Hakan Ozkan, Gencehan Kumtepe, Ilker H Karal, Serdar Ener

**Affiliations:** Department of Cardiovascular Surgery, Balikesir University, School of Medicine, Balikesir, 10010 Turkey; Department Of Cardiology, Bahcesehir University Faculty of Medicine, Istanbul, Turkey; Department of Cardiovascular Surgery, Samsun Hospital for Education and Research, Samsun, Turkey; Department of Cardiovascular Surgery, Acıbadem Bursa Hospital, Bursa, Turkey

**Keywords:** Paravalvular leak, Percutaneous catheter ablation, Prosthetic heart valve

## Abstract

An increasing number of reports reveal the safety and efficacy of catheter ablation for atrial fibrillation therapy even in patients with a history of prosthetic heart valve. This case report presents a 60 year old female patient who had a prosthetic mitral paravalvular leak as a complication of radiofrequency ablation. Surgery revealed a 15 mm hole and three broken knots in the posterolateral mitral annulus. This report demonstrates radiofrequency ablation for atrial fibrillation in patients with prosthetic mitral valve may cause a paravalvular leak likely as a result of thermal injury. Moreover, patient can be asymptomatic at first. The clinician should keep in mind such complication and the patient should be evaluated in terms of paravalvular leakage.

**Electronic supplementary material:**

The online version of this article (doi:10.1186/s13019-014-0187-4) contains supplementary material, which is available to authorized users.

## Background

Since its first description in 1982 [1], radio frequency catheter ablation (RFA) has evolved to its present role as first-line therapy for most supraventricular arrhythmias [2]-[6]. The growing acceptance of this procedure has been brought about by an increasing number of reports revealing the safety and efficacy of catheter ablation for atrial fibrillation (AF) therapy. Reported complications of RFA include stroke, pericardial tamponade, valvular injury, pulmonary embolism, atrio-esophageal fistula and significant pulmonary vein (PV) stenosis [4]-[6]. We report the case of a patient with a history of mechanical mitral valve replacement (MVR) who had a paravalvular leak as a complication of RFA for AF therapy. This case is presented in order to draw attention to this rare presentation which has never been previously reported in the medical literature.

## Case presentation

**Figure 1:**
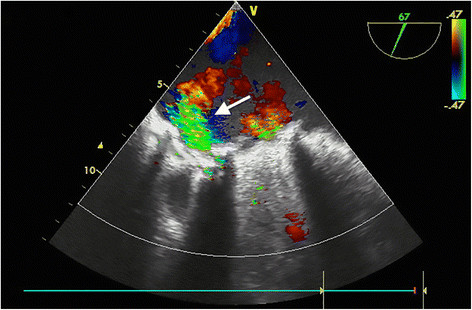
**Transesophageal echocardiography illustrating paravalvular mitral regurgitation.** Arrow indicates paravalvular leakage.

**Figure 2:**
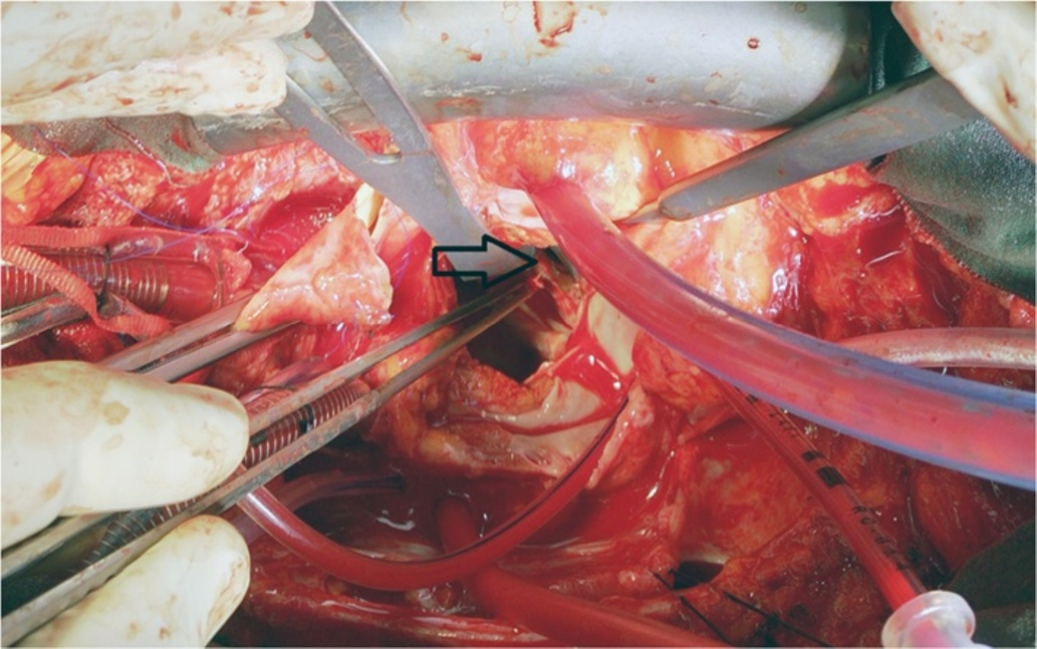
**Perioperative image of paravalvular leak area.** Arrow indicates paravalvular leakage area.

**Figure 3:**
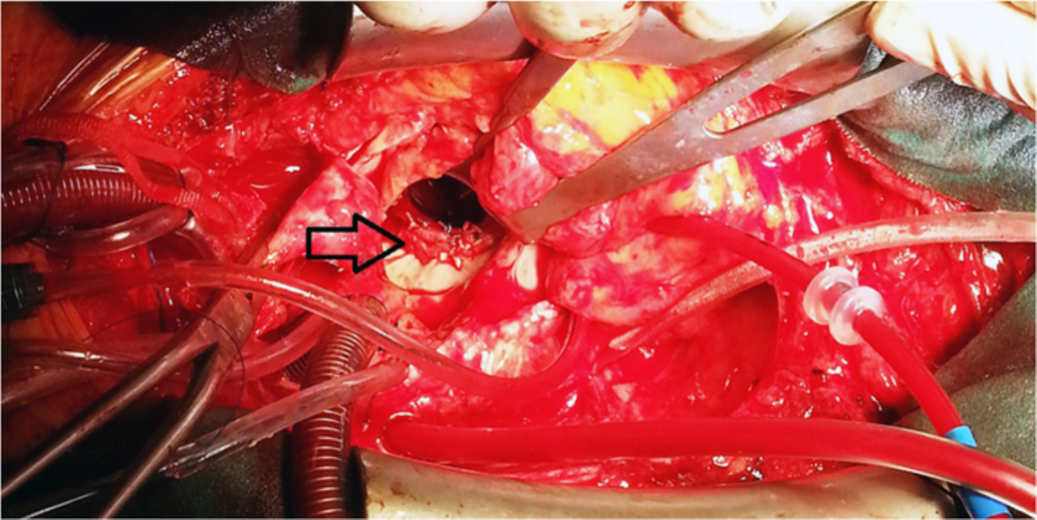
**Periopertive image of repaired paravalvular leak area.** Arrow indicates repaired paravalvular leakage.

A sixty-year-old woman with the posterolateral prosthetic paravalvular leak (PVL) was referred to our clinic for surgical repair. She had a history of closed mitral commissurotomy 47 years ago, a prosthetic mitral valve replacement (MVR) in 1975 and a redo MVR in 2005 due valve dysfunction by pannus formation*.* Moreover, 1 years ago, she had undergone a pulmonary vein isolation (PVI), left atrium and mitral isthmus roof lines ablations for symptomatic persistent AF in another hospital, using CARTO mapping system (Biosense, Diamond Bar, CA, USA) and an irrigation ablation catheter (Biosense, ThermoCool® SF Catheter, USA). Furthermore, RFA was delivered at pulmonary veins for up to 30 W and at both lines up to 35 W with a temperature limitation of 50°C. Despite successful conversion to sinus rhythm she developed progressive cardiac failure in months. Third month later, after the percutaneous ablation, she was classified as decompensating heart failure in New York Heart Association (NYHA) stage four. Transthoracic echocardiography revealed a moderate PVL with an ejection fraction of 60%. Transesophageal echocardiography (TEE) demonstrated moderate to severe posterolateral prosthetic PVL (Figure 1). She was referred for surgery as she refused percutaneous closure of PVL. This patient was under echocardiography follow-up every three months, which were not revealed any PVL or pulmonary hypertension before the procedure, but three months after the RFA. After hospitalization, she underwent a coronary angiography, which revealed no obstructive lesions in coronary arteries. Following a full-resternotomy, pericardial adhesions were separated by sharp dissection, then cardiopulmonary bypass was initiated by aortic and bicaval venous cannulation. Systemic temperature was actively cooled down to 32°C. Following aortic cross-clamping, myocardial protection was achieved with antegrade and retrograde cold blood cardioplegia. The left atrium was reached by transseptal approach. A 15 mm defect was detected between mechanical valve and posterolateral mitral annulus where knot of 3 sutures was found to be broken (Figure 2). The defect was successfully repaired using separated 2-0 pledgeted sutures (Figure 3). İmmediately before declamping hot shot solution was administered. After declamping the heart was in nodal rhythm, therefore 5 mcg/kg/min dopamine infusion and VVI pacing via 2 epicardial pacing leads was initiated. Cardiopulmonary bypass ended 50 minutes after its initiation. İntraoperative control TEE did not reveal any PVL and patient was transferred to the intensive care unit (ICU). She was extubated within 12 hours and dopamine infusion was stopped. On the second postoperative day, normal sinus rhythm returned and she was transferred out of the ICU. On the fourth postoperative day atrial fibrillation occurred which was converted into sinus rhythm by amiodarone. The patient was successfully discharged on the 7th day postoperatively in sinus rhythm and completed the 1*-* year follow*-* up without complications.

## Discussion

Atrial fibrillation is the most common cardiac rhythm disturbances, with a prevalence estimated at 0.5% of the population [7]. It frequently arises from an abnormal focus near the junction of the pulmonary veins and the left atrium.

RFA is a widely used technique as a first-line therapy for a variety of supraventricular arrhythmias with a low incidence of complication. Studies generally report short-term recurrence rates up to 30%, requiring repeat ablations. Moreover, if early recurrence does not occur longer-term recurrence rates are reported lower, in the range of 1-2% per year [8],[9]. American Heart Association (AHA) suggests catheter ablation in patient with symptomatic paroxysmal AF or selected patients with symptomatic persistent AF [10].

Serious complications, including cardiac tamponade, pulmonary embolism, inferior myocardial infarction, significant PV stenosis or PV thrombosis, valvular injury, atrio-esophageal fistula and stroke are likely in the 1%–5% range.

Our report reveals PVL as a complication of this procedure in a patient with recent prosthetic mitral valve replacement. PVL is a well-known complication of prosthetic valve implantation, with an estimated incidence of 3% to 6% [11]. PVL can be asymptomatic or can have significant clinical consequence, such as congestive heart failure, hemolytic anemia, and infective endocarditis. Clinical success rates of percutaneous PVL repair approach 80% to 90% in selected patients [11].

Recent reports revealed favorable outcomes of RFA for atrial fibrillation therapy in patients with previous MVR. Moreover, PVL has never been previously reported after RFA [12],[13].

Our report shows that during the RFA of left atrium, suture knots can be damaged by catheter induced thermal injury and PVL might develop. Interestingly, this complication has not been noticed immediately after treatment, but just after the development of heart failure.

## Conclusion

Radiofrequency ablation for atrial fibrillation in patients with prosthetic mitral heart valve may cause a paravalvular leak which can be asymptomatic at first. The clinician should keep in mind such complication and the patient should be evaluated in terms of paravalvular leakage.

## Consent

Written informed consent was obtained from the patients for publication of this case report and all accompanying images.

## Authors’ original submitted files for images

Below are the links to the authors’ original submitted files for images. Authors’ original file for figure 1 Authors’ original file for figure 2 Authors’ original file for figure 3

## Abbreviations

AF: Atrial fibrillation
AHA: American heart association
ICU: Intensive care unit
MVR: Mitral valve replacement
NYHA: New york heart association
PVL: Paravalvular leak
PV: Pulmonary vein
RFA: Radio frequency catheter ablation
TEE: Transesophageal echocardiography
