# Supplementary figures and images for: Case report: paravalvular leak as a complication of percutaneous catheter ablation for atrial fibrillation
Source: J Cardiothorac Surg. 2014 Dec 17;9:187. doi: 10.1186/s13019-014-0187-4 (PMC4279701; doi:10.1186/s13019-014-0187-4)

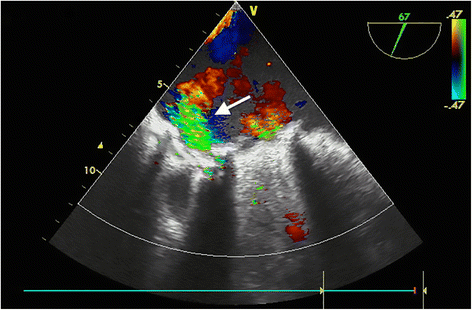

Supplement: Supplementary file 1 — Authors’ original file for figure 1 [file 13019_2014_187_MOESM1_ESM.gif]

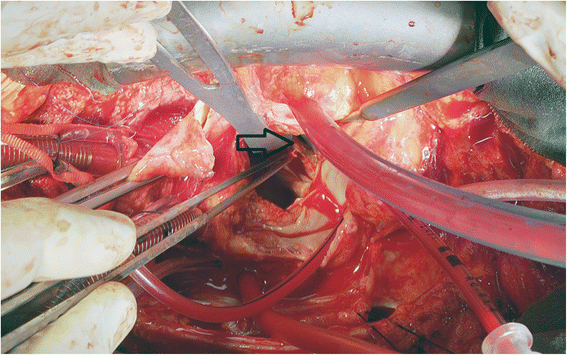

Supplement: Supplementary file 2 — Authors’ original file for figure 2 [file 13019_2014_187_MOESM2_ESM.gif]

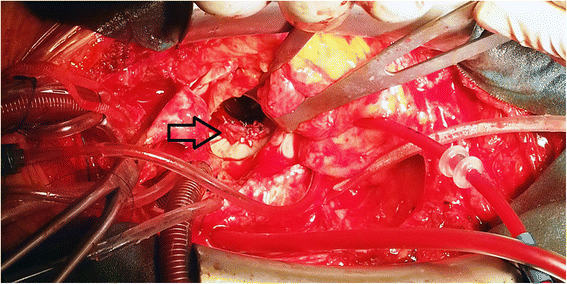

Supplement: Supplementary file 3 — Authors’ original file for figure 3 [file 13019_2014_187_MOESM3_ESM.gif]
